# The Waste of Brain Resources

**Published:** 1888-07-28

**Authors:** 


### The Doctor's Pulpit.

### THE WASTE OF BRAIN RESOURCES.

Few people who live by their brains feel conscious of superfluous mental power. They rather experience a keen and sometimes even an angry sense of impotence and ineffectuality. They have things to say and teach which the world not merely needs to learn, but is seriously injured by not learning. Yet nobody, or only a very few, care a straw for their "lessons in the science and art of living." "Ever," said Carlyle, "to be weak is the true misery." It is one thing, however, to be weak, and quite another to be regarded as weak, although abundance of strength be present. Many a man is of little account in the world because he is not of a self-asserting disposition. Such a man was John Stuart Mill, and had he been left to indulge his native modesty, the world would probably have been the poorer by the want of all the true and just things he has said.

There are two classes of persons who may be said to largely waste the resources of intellectual and moral wealth with which Nature has endowed them—these are the modest and the cowardly. With the modest man it is difficult to find fault. Compared with the unintellectual myriads of his fellow-men, he does not belong to a numerous class. But the shrinking from conflict and contention, which is a common habit of quiet-loving mortals, is often very injurious: injurious to the great world, injurious to loud-voiced and comparatively feeble-brained opponents, and injurious to the modest man himself. Experience and reflection seem to say certainly that the world was meant to be a place of struggle, a scene of conflict. Intellect is to strive with stupidity, and to overthrow it; reason is to wrestle with prejudice; truth is to do manful battle, not only against that which is not true, but also for its own establishment and permanence. The opponent will often be self-asserting, sometimes offensive, and not seldom vulgar. His only object will seem to be to make his point. A reason is to him of no moment if it is advanced by the opposite party. However sound the reason may be, he will make believe to blow it away with contempt. Or he may be flippant, and acknowledge no moral standard. He will raise some small and inconsequent point in order to get the laugh against his adversary, and so appear to win a victory when he has not even crossed swords.

Now, the man who takes life with reasonable seriousness feels inclined to regard such opponents as these with contempt. "What is the use of putting pearls into a pig's trough?" he asks himself. He therefore often hides his candle of honest and truthful intent out of sight, and only uncovers it when he thinks the moment favourable.

That is not the right way to do. The world is constantly in need of all the brains and the conscience it can get. What sort of a horrid stagnant mass would the seas and oceans become if the water were deprived of its salts? Just such a pestilent and destructive chaos would society be if its pure reason and conscience should disappear. It is the duty of the modest man who has brains and conscience always to give those faculties their utmost degree of effect on all occasions. The empty boaster must be met by reason, and silenced; the crafty propagator of half truths, untruths, and false principles must be tracked out and exposed. This duty the modest man owes to himself, to the world, and to the Power, whatever name it be called by, who endowed him with the high faculties of pure and unprejudiced reason and an honourable conscience. Failing the performance of the duty, those brain resources, which the world needs more than even its material wealth, are to a large extent wasted. Moreover, the faculties tend to atrophy for want of use.

But we wish, at the present time, to make our contention more especially with him who, possessing brains and culture, fails to give effect to the conclusions of his intellect from pure cowardliness. Small states, in the dawn periods of their progress, oftener give rise to great men than large empires and old civilisations. Why should that be? General culture is more advanced, and there are ten or twenty times the number of possible

Homers or village Hampdens in the later epochs. One reason is that in a large state men cannot see everything that is to be seen with their own eyes, and therefore cannot take the just measure of the large as they can of the small state. The mere sense of numbers and of vastness oppresses them, and makes them think achievement impossible. Among a few people, and in a small community, they could see and know everyone and everything of moment, and therefore were not afraid to advance their opinions. The really practical and capable man under such circumstances sooner or later came to the front without elbowing his way there. In these later times it is not capacity which always comes to the front, but confidence—self-confidence. A man has the wish to stand in the first rank, and coupled with the wish he has the hardihood to try. Courage, therefore, or, if it please better, self-confidence alone, unaccompanied by any other great quality, will frequently carry a man far ahead of many of his fellows who in a thousand other ways are infinitely his superiors.

Now shall we blame the courage of the one man which takes him ahead, or shall we not rather say the fault lies with other men in that their cowardice leaves them in the rear? The high and difficult mountains of life lie before all men. Each is called to scale them, and to reach such elevation as may be possible. It is to the world's interest and advantage that the strongest, the wisest, and the most virtuous shall gain the highest point. It frequently happens that the strongest only is successful; but that would not so often be the case if the wise and virtuous made up for their smaller strength by their greater courage and resolution. That they often fail to do, and consequently find themselves, when their powers ought to be at their best, solving problems which should have been solved twenty years before, and doing the tasks of boys rather than those proper to ripe manhood.

The great and main cause of intellectual feebleness and waste is the failing to give effect in action to convictions gained from experience or from books. A man frequently finds himself at different periods of his life at the beginning of two ways, one of which he sees to be the better and the nobler. That way he would choose if he were left to follow absolutely his own generous instincts and pure reason. But other persons and circumstances enter into his calculations, and the less worthy way is chosen. That kind of conduct is fatal to progress and to intellectual and moral growth. It is fatal, not only to the individual but to the community. It is thus that so much of the splendid originality of this country is entirely wasted and thrown away. There seems to be a conspiracy on the part of routine and interested persons to choke and destroy all that is simple, natural, and straightforward in their fellows. The conspirators are legion, and one by one they draw over to their ranks the majority of those who, if they had courage equal to their other qualities, would continually renew the youth and the vigour of the world. It is thus that not merely moral character is deteriorated, but brain-power is steadily diminished and enfeebled. Just as the muscles of the arms and legs shrink and atrophy for want of due exercise, so does the brain lose its vigour and effectiveness by failing to constantly carry into action the deeper convictions which life, experience, and weighty books cannot fail to fashion in the mind. Thus are the light and strength which should be spent in the development of the race wasted and destroyed in fighting over again old intellectual battles which have already been fought a hundred times, and in playing at problems which a sterner purpose would have solved and forgotten long ago. Thus is a man of middle age still a child for all practical purposes, and will continue so until his second childhood finally ends his possibilities. This is eminently a doctor's question. Probably no one sees so clearly, in the steady loss of brain-power, the evil which results from the stifling of earnest convictions. Life must be either trifling or serious. If it be trifling, the man who lives it becomes imbecile; if it be serious, he increases in intellectual strength and capacity so long as his physical energy endures.

